# Systematic evaluation of particle loss during handling in the percutaneous transluminal angioplasty for eight different drug-coated balloons

**DOI:** 10.1038/s41598-020-74227-1

**Published:** 2020-10-14

**Authors:** Andreas Heinrich, Martin S. Engler, Felix V. Güttler, Christian Matthäus, Jürgen Popp, Ulf K.-M. Teichgräber

**Affiliations:** 1 Department of Radiology, Jena University Hospital-Friedrich Schiller University, Am Klinikum 1, 07747 Jena, Germany; 2 Leibniz Institute of Photonic Technology, 07745 Jena, Germany; 3 Institute of Physical Chemistry & Abbe Center of Photonics, Friedrich Schiller University Jena, 07743 Jena, Germany

**Keywords:** Translational research, Mechanical engineering, Polarization microscopy

## Abstract

Paclitaxel drug coated balloons (DCBs) should provide optimal drug transfer exclusively to the target tissue. The aim of this study was to evaluate the particle loss by handling during angioplasty. A robotic arm was developed for systematic and reproducible drug abrasion experiments. The contact force on eight different commercially available DCB types was gradually increased, and high-resolution microscopic images of the deflated and inflated balloons were recorded. Three types of DCBs were classified: no abrasion of the drug in both statuses (deflated and inflated), significant abrasion only in the inflated status, and significant abrasion in both statuses. Quantitative measurements via image processing confirmed the qualitative classification and showed changes of the drug area between 2.25 and 45.73% (13.28 ± 14.29%) in the deflated status, and between 1.66 and 40.41% (21.43 ± 16.48%) in the inflated status. The structures and compositions of the DCBs are different, some are significantly more susceptible to drug loss. Particle loss by handling during angioplasty leads to different paclitaxel doses in the target regions for same DCB types. Susceptibility to involuntary drug loss may cause side effects, such as varying effective paclitaxel doses, which may explain variations in studies regarding the therapeutic outcome.

## Introduction

Percutaneous transluminal angioplasty (PTA) is a minimally invasive endovascular procedure aimed at widening narrowed or obstructed blood vessels^1^. For this purpose, a catheter with an attached deflated balloon is passed over a sheath and guide-wire into the narrowed vessel and then inflated to a fixed size. Additionally, a stent may be inserted to ensure that the vessel remains open. After improvement of the blood flow by expansion of the blood vessel and the surrounding muscular wall, the balloon is then deflated and withdrawn. One disadvantage is that PTA is more prone to restenosis than vascular bypass or coronary artery bypass grafting^2,3^. Drug-eluting balloon (DCB) angioplasty, due to the prevention of mitosis, involves significantly less restenosis than non-coated plain balloon angioplasty^4^. For currently used DCBs, paclitaxel represents the most often used drug that is provided with manufacturer-specific coatings in different concentrations and with different excipients on the balloon surface. DCB is a promising emerging technology^5^ following the “leaving nothing behind” principle^6^ and providing favorable initial results in areas where a drug-eluting stent (DES) is not suitable. Nevertheless, restenosis remains a major issue in endovascular treatment^7^. The recommended treatment of restenosis is repeat revascularization of the target lesions, target vessels, or non-target vessels^8^. However, in some cases this results in a high number of repeated treatments^9^, which emphasizes the need for devices with a low restenosis risk.

A hypothesis to explain restenosis after DCB and DES treatments is involuntary particle detachment outside the target lesion due to difficult device delivery, leading to non-uniform drug distribution at the target site^4,10^. The coating of paclitaxel DCB for targeted drug delivery is subject to an inherent conflict of objectives. On the one hand, the adherence of the drug to the excipient is weak, so the drug is easily transfer to the tissue of the target region after therapeutic balloon inflation. However, that renders the DCB inherently vulnerable to involuntary particle detachment. On the other hand, the drug can adhere strongly to the excipient, so that there is only limited drug loss during transport. However, that may result in limited drug transfer at the target area^11^.

The recent literature^12–16^ reports several methods for measuring the loss of paclitaxel. All methods proposed treat the particle loss by handling during angioplasty as a black box. The concentration of the drug was measured by high performance liquid chromatography (HPLC) before and after stress on the balloon surface. Angiography procedures can differ widely, depending, for example, on the target region and the experience of the physician. Furthermore, only small groups of commercial DCBs have been examined, and only a few publications have compared different DCB types with each other. For this purpose, a new systematic and reproducible method to evaluate the particle loss by handling during angioplasty for a wide range of currently used DCBs was developed and applied.

## Results

**Figure 1:**
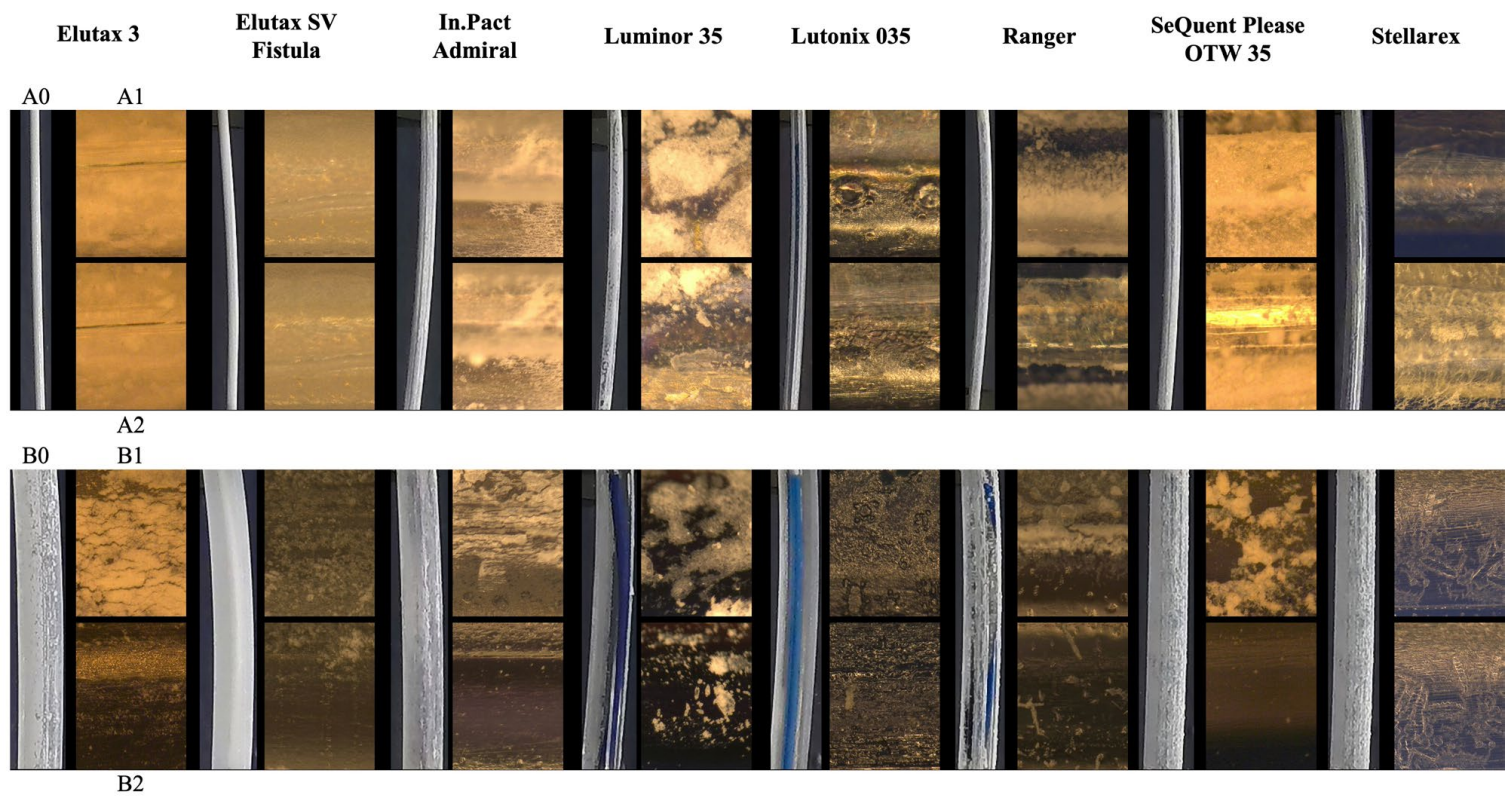
Photographs of the DCBs for a deflated (**A0**) and an inflated (**B0**) status, and microscopic images magnified ×200 before (**A1**, **B1**) and after (**A2**, **B2**) the abrasion process for a deflated (**A**) and an inflated (**B**) DCB.

The DCBs have a diameter between 1.20 and 2.01 mm in the folded (deflated) status (see Table 1). The nominal diameter of the inflated balloon is 5 mm for all investigated DCBs. The drug distribution and coating technologies of the DCBs are varied (see Fig. 1A0,B0). When unpacking and removing the protective cap, we partially observed a minor loss of drug/excipient for the Luminor 35 and SeQuent Please OTW 35. When inflated, the drug was distributed in stripes on the surface of the balloon for the Luminor 35 and Ranger. For the other DCBs, the drug was evenly distributed over the surface, however partly with gaps (spots, compare with Fig. 1).

**Table 1:** Qualitative description of the drug loss in deflated and inflated status.

Name	DCB deflated		DCB inflated	Class
	Characteristics	Diameter (mm)	Characteristics	
Elutax 3	No or hardly any abrasion of the drug/excipient	1.20	Clear abrasion of the drug/excipient	1
Elutax SV Fistula		1.34		1
In.Pact Admiral		2.01		1
Luminor 35	Clear abrasion of the drug/excipient	1.94		2
Lutonix 035	No or hardly any abrasion of the drug/excipient	1.98	Drug shifted slightly, hardly any abrasion of the drug/excipient	0
Ranger	Clear abrasion of the drug/excipient	1.50	Clear abrasion of the drug/excipient	2
SeQuent Please OTW 35		1.92		2
Stellarex	Clear structure change inside, but no abrasion of drug/excipient	1.96	Drug shifted slightly, hardly any abrasion of the drug/excipient	0

Additionally, the measured diameter for the deflated DCBs is shown.

### Qualitative evaluation

**Figure 2:**
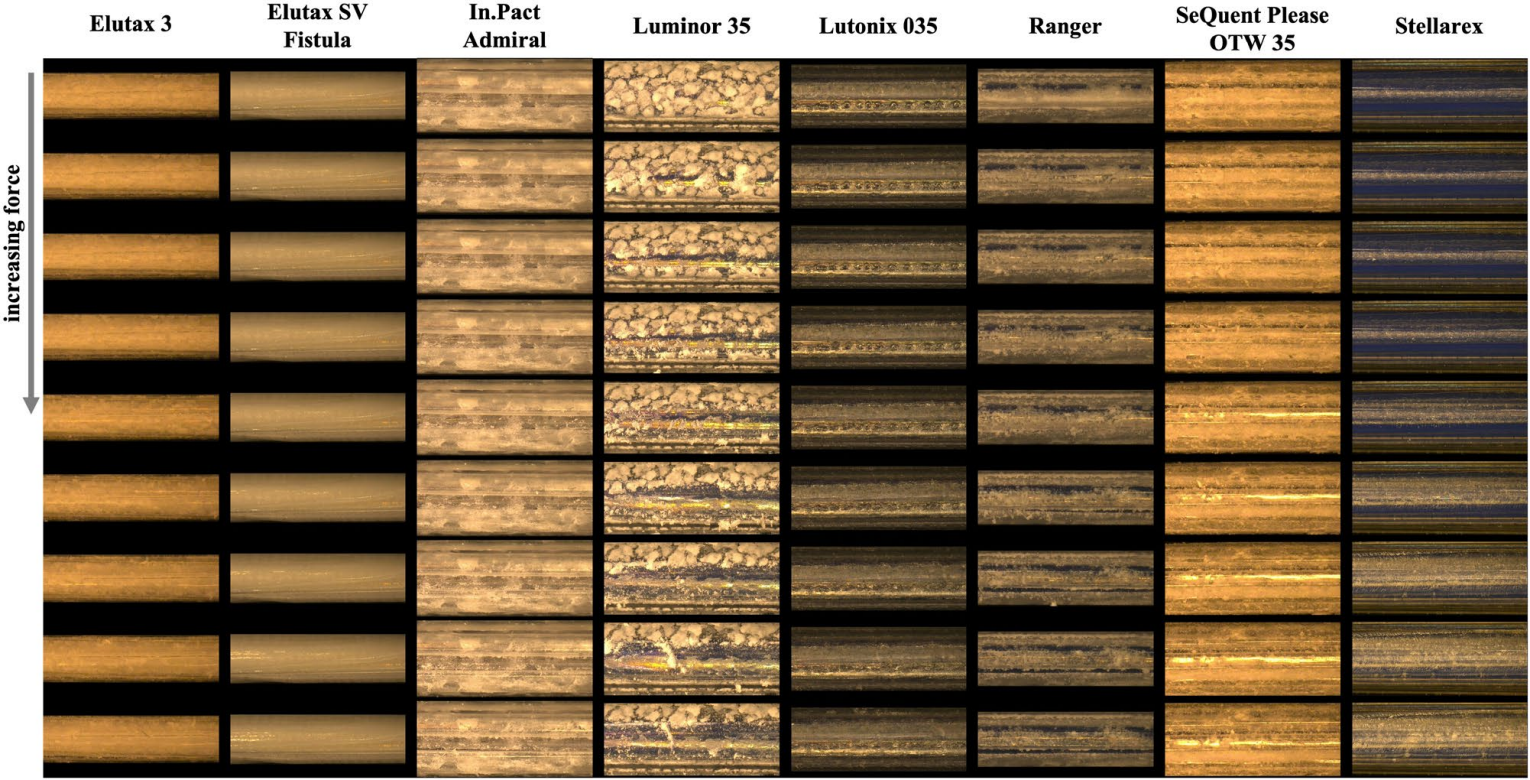
Microscopic images magnified ×50, recorded during the abrasion process for the deflated DCBs. The Stellarex represents a special case, where the lightness changes are caused by internal structure changes.

**Figure 3:**
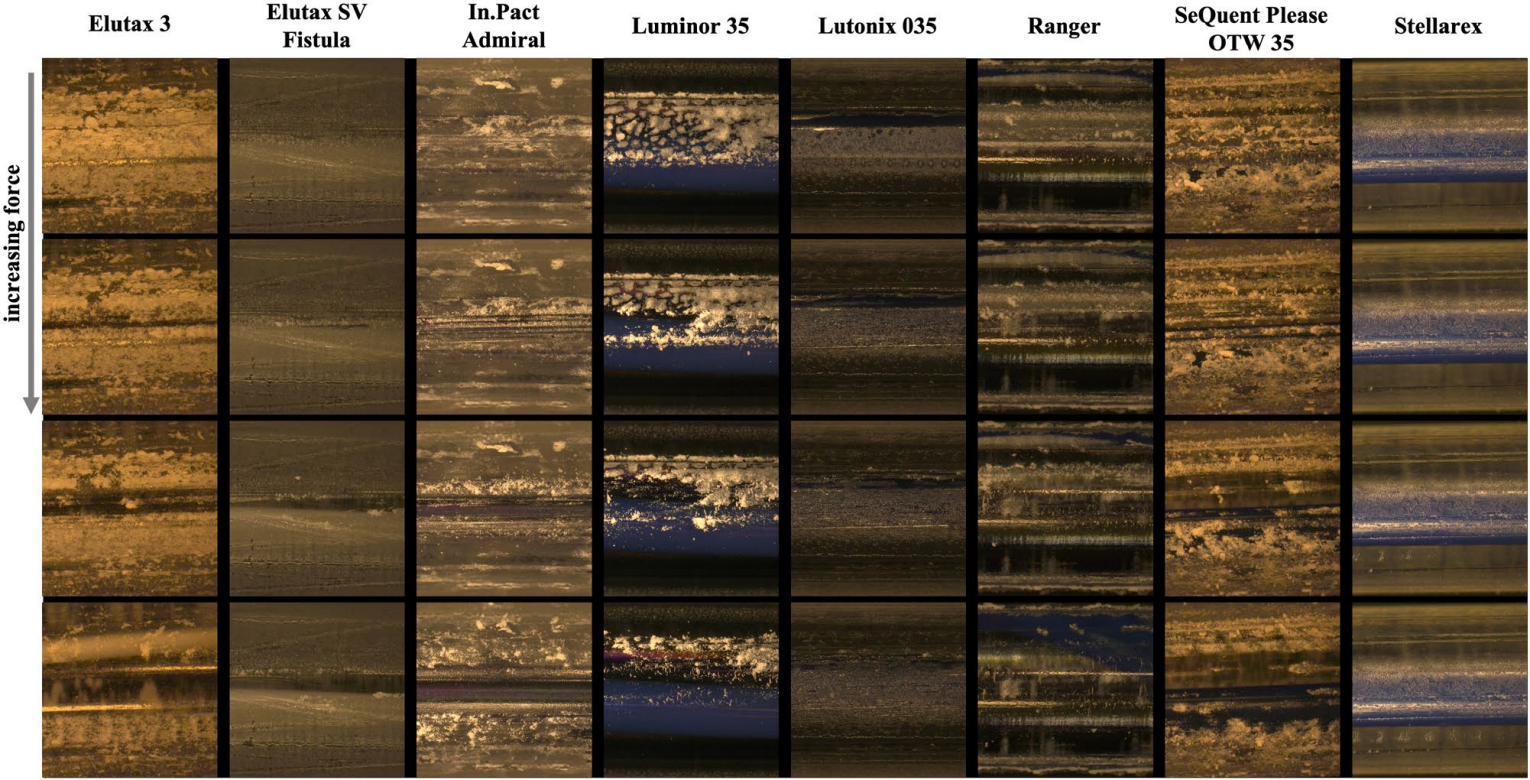
Microscopic images magnified ×50, recorded during the abrasion process for the inflated DCBs.

Three types of DCBs were classified (see Figs. 2, 3). Two DCBs (Lutonix 035 and Stellarex) showed no or hardly any abrasion of the drug in both statuses (classification 0). Three DCBs (Elutax 3, Elutax SV Fistula, In.Pact Admiral) showed significant abrasion of the drug only in the inflated status (classification 1). Three DCBs (Luminor 35, Ranger, SeQuent Please OTW 35) suffered from significant abrasion of the drug in both statuses (classification 2). With a significant abrasion of the drug, the balloon surface was completely rubbed off, and only the transparent balloon envelope remained.

### Quantitative evaluation

**Figure 4:**
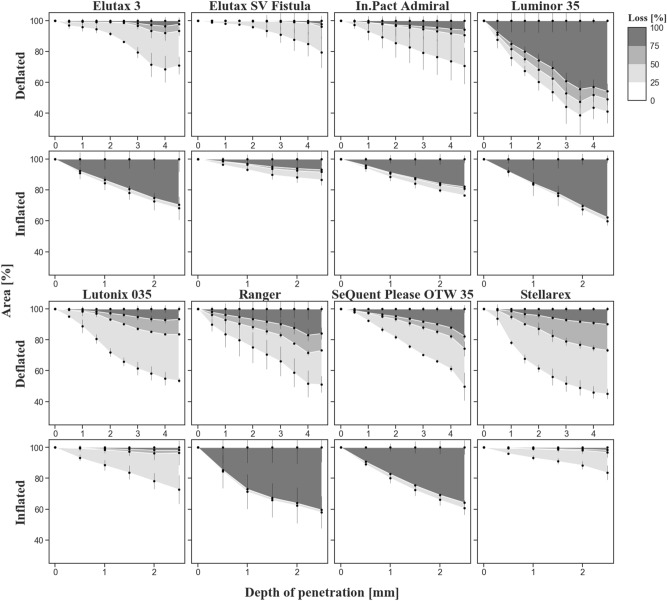
Stacked area chart of the percentage of loss of lightness in the drug area for each deflated and inflated balloon. The depth of penetration of the abrasion blade was gradually increased by reducing the height between balloon surface and abrasion blade. High percentages of losses (dark gray area) indicate significant drug losses, while low losses (light gray area) may be due to actual minor drug losses and/or image noise. The deflated Stellarex represents a special case, where the lightness changes are caused by internal structure changes.

The quantitative results confirm the qualitative classification (see Fig. 4 and Supplementary Material Figs. S1–S8). Image registration corrected the image shifts well despite significant image changes due to large drug losses. The reflection suppression also filtered an artifact caused by a moving internal guide wire tube for the Elutax 3. High percentages of losses (dark gray area) indicate significant drug losses, which was confirmed by visual inspection of the images. However, low losses (light gray area) may be due to both actual minor drug losses and image artifacts, including illumination changes, reflections and/or minor image shifts. The depth of penetration, and therefore also the contact force of the abrasion blade, was gradually increased by reducing the height between balloon surface and abrasion blade by 0.5 mm after each cycle until it reached 4.50 mm and 2.50 mm for the deflated and inflated balloons, respectively. Under the same conditions, significant differences between the DCB types were found (Table 2). In the deflated status (see Fig. 4 Deflated), virtually no change of the drug area was observed for the Elutax 3 and Elutax SV Fistula with a loss of 100% lightness (dark gray area) in 2.31 ± 2.03% and 2.25 ± 0.70% of the drug area at increasing contact force up to 0.94 ± 0.28 N and 1.51 ± 1.12 N, respectively. A minor change of the drug area (> 5% loss of 100% lightness) was observed for the In.Pact Admiral and Lutonix 035 with a loss of 100% lightness in 5.95 ± 4.83% and 6.65 ± 1.58% at increasing contact force up to 1.83 ± 0.59 N and 2.21 ± 0.05 N, respectively. In contrast, a significant change of the drug area (> 10% of loss of 100% lightness) was observed for the Luminor 35, Ranger, and SeQuent Please OTW with a loss of 100% lightness in 45.73 ± 0.85%, 15.82 ± 1.92%, and 17.62 ± 2.50% of the drug area at increasing contact force up to 1.75 ± 0.16 N, 1.34 ± 0.09 N, and 2.22 ± 0.17 N respectively. The Stellarex shows a special behavior: visual inspection of the images showed there is no loss of the drug, but a clear structure change (small breaks in the layer) inside the DCB, resulting in a loss of 100% lightness in 9.87 ± 1.42% of the drug area at increasing contact force up to 2.77 ± 0.59 N.

**Table 2:** The loss of 100% lightness (compare with dark gray area in Fig. 4) of DCB types were compared by the independent-samples Kruskal–Wallis test with post hoc Dunn’s pairwise tests and Bonferroni corrections.

	P value (effect size)							
	Elutax 3	Elutax SV Fistula	In.Pact Admiral	Luminor 35	Lutonix 035	Ranger	SeQuent Please OTW 35	Stellarex
**DCBs deflated**								
Elutax 3		1.000	1.000	** < 0.001 (large)**	0.267	** < 0.001 (large)**	** < 0.001 (large)**	**0.013 (medium)**
Elutax SV Fistula	1.000		0.098	** < 0.001 (large)**	**0.003 (large)**	** < 0.001 (large)**	** < 0.001 (large)**	** < 0.001 (large)**
In.Pact Admiral	1.000	0.098		** < 0.001 (large)**	1.000	0.105	0.116	1.000
Luminor 35	** < 0.001 (large)**	** < 0.001 (large)**	** < 0.001 (large)**		** < 0.001 (large)**	**0.032 (medium)**	**0.024 (medium)**	**0.001 (large)**
Lutonix 035	0.267	**0.003 (large)**	1.000	** < 0.001 (large)**		1.000	1.000	1.000
Ranger	** < 0.001 (large)**	** < 0.001 (large)**	0.105	**0.032 (medium)**	1.000		1.000	1.000
SeQuent Please OTW 35	** < 0.001 (large)**	** < 0.001 (large)**	0.116	**0.024 (medium)**	1.000	1.000		1.000
Stellarex	**0.013 (medium)**	** < 0.001 (large)**	1.000	**0.001 (large)**	1.000	1.000	1.000	
**DCBs inflated**								
Elutax 3		**0.024** (**large**)	1.000	1.000	** < 0.001 (large)**	1.000	1.000	** < 0.001 (large)**
Elutax SV Fistula	**0.024 (large)**		0.680	**0.002 (large)**	1.000	**0.001 (large)**	**0.001 (large)**	1.000
In.Pact Admiral	1.000	0.680		1.000	**0.033 (large)**	0.217	1.000	**0.025 (large)**
Luminor 35	1.000	**0.002 (large)**	1.000		** < 0.001 (large)**	1.000	1.000	** < 0.001 (large)**
Lutonix 035	** < 0.001 (large)**	1.000	**0.033 (large)**	** < 0.001 (large)**		** < 0.001 (large)**	** < 0.001 (large)**	1.000
Ranger	1.000	** < 0.001 (large)**	0.217	1.000	** < 0.001 (large)**		1.000	** < 0.001 (large)**
SeQuent Please OTW 35	1.000	**0.001 (large)**	1.000	1.000	** < 0.001 (large)**	1.000		** < 0.001 (large)**
Stellarex	** < 0.001 (large)**	1.000	**0.025 (large)**	** < 0.001 (large)**	1.000	** < 0.001 (large)**	** < 0.001 (large)**	

The effect sizes (r) were divided into small (r < 0.3), medium (0.3 ≤ r ≤ 0.5) and large (r > 0.5). The level of significance was set to p < 0.05, and significant P values are shown in boldface.

In the inflated status (see Fig. 4 Inflated), Elutax 3, In.Pact Admiral, Luminor 35, Ranger, and SeQuent Please OTW 35 show a significant change of the drug area (> 10% of loss of 100% lightness) with a 100% lightness loss in 29.52 ± 8.15%, 17.90 ± 2.41%, 37.73 ± 2.44%, 40.41 ± 9.55%, 35.70 ± 4.61% of the drug area at increasing contact force up to 1.94 ± 1.02 N, 1.64 ± 0.18 N, 1.68 ± 0.22 N, 1.30 ± 0.30 N and 1.72 ± 0.15 N, respectively. Elutax SV Fistula shows a minor change of the drug area (> 5% of loss of 100% lightness) with a 100% lightness loss in 6.85 ± 4.78% of the drug area at increasing contact force up to 1.64 ± 0.19 N. The Lutonix 035 and Stellarex show virtually no loss of the drug with a 100% lightness loss in 1.70 ± 1.00% and 1.66 ± 0.48% of the drug area with increasing contact force up to 1.90 ± 0.26 N and 2.15 ± 0.27 N, respectively. The error bars correspond to the standard deviation caused by the measurement uncertainty due to repeated measurements and a partial shift in the measurement area due to the force effect, which could not always be corrected completely.

## Discussion

The DCB types differ with regard to the coating technology, drug dose and the excipient (Table 3), which is why different responses to frictional force were observed. Fundamentally important during drug delivery by DCBs are the following desired properties. First, homogeneous and consistent drug delivery to the lesion^11^. Second, not losing too much drug and excipient during insertion of the catheter into the sheath or before reaching the lesion^17^. Third, releasing a sufficient amount of drug into the vessel wall during inflation and a prolonged delivery of sufficiently high levels of paclitaxel to reduce smooth muscle cell proliferation and vessel restenosis^18–21^. The drug dose, coating and type of excipient can have an impact on restenosis reduction and clinical outcomes^4,22–24^. For example, highly crystalline coatings are more likely to cause distal embolization due to particle depots with higher and longer tissue residency time on the vessel wall and low solubility. The excipient modulates drug transfer into the vessel wall^25,26^.

**Table 3:** Summary of investigated DCBs with a length of 40 mm and a diameter of 5 mm.

Manufacturer	Name	REF (LOT)	Paclitaxel dose (µg/mm^2^)	Excipient	Nominal pressure (bar)
Aachen Resonance GmbH	Elutax 3	102540 (Elutax 3)	2.0	Dextran	6.0
	Elutax SV Fistula	ELUTAX-SV-OTW-S40500 (AR291119-EC)	2.0	None	6.0
Medtronic	In.Pact Admiral	SBI05004008P (0010076231)	3.5	Urea	8.1
iVascular	Luminor 35	BPDPC35080500040 (1910747)	3.0	Organic ester	7.1
BD-Interventional	Lutonix 035	9090475500040 (GFDR0210)	2.0	Polysorbate and sorbitol	6.1
Boston Scientific Corporation	Ranger	H74939219500480 (13414H19)	2.0	Acetyl tributyl citrate	6.0
B. Braun Melsungen AG	SeQuent Please OTW 35	35150040 (19L22844)	3.0	Resveratrol	6.0
Philips	Stellarex	A35SX050040080 (F2B19B12A)	2.0	Polyethylene glycol	10.1

For Elutax 3, Elutax SV Fistula, In.Pact Admiral, Lutonix 035 and Stellarex, the drugs are folded inwards and/or the drug layer gets protected by a top coating or hydrophilic coating, so that the drug suffers only minor losses despite any large frictional force in the sheath and vascular system. This property is desirable to ensure that the drug reaches the target region completely. In contrast, for Luminor 35, Ranger and SeQuent Please OTW 35, the drug is partly abraded in the sheath or the vascular system before it reaches the target region. Especially for the Luminor 35, any contact results in drug loss, whereas the other two DCBs withstand low frictional forces. For the Luminor 35 and Ranger, the drug seems to be sprayed only onto the outside of the folded balloon, which is why after inflation the distribution is only visible in stripes. Another important property is the delivery of the drug to the target region. This study showed that all DCBs, except Lutonix 035 and Stellarex, deliver the drug particularly easily by friction on the vessel wall. However, this process is not automatically comparable to higher transfer of drugs to the vessel wall. Differences can be caused by the excipient intended to optimize paclitaxel microcrystallinity^27^. For Stellarex and Lutonix 035 it can be seen that the excipient binds the drug very strongly for a slower dissolution rate^23,28^ and that, even with the greatest force, hardly any drug is released.

In the folded state, the diameter of the DCB is manufacturer-specific and depends, for example, on the excipient and drug dose. With a larger diameter, more frictional force can act on the DCB, and more drug may be lost during passage until it reaches the lesion. Elutax 3 with Dextran and Elutax SV Fistula without an excipient showed the smallest diameters. The TransPax coating of Ranger with drug applied outside of the folded balloon also leads to a small diameter. In contrast, the diameter of the Luminor 35 is larger, most likely caused by its high paclitaxel dose. Although the drug is partially folded inwards, the SeQuent Please OTW 35 has a diameter comparable to that of Luminor 35 with a comparable dose, which may be due to the excipient used. Despite a low paclitaxel dose, the Lutonix O35 and Stellarex have relatively large diameters, which is probably due to the excipient and/or the coating technology. In.Pact Admiral shows the largest diameter, but here the paclitaxel dose is particularly high, and the drug is additionally folded inwards.

The recent literature^12–16^ reports several methods for measuring the loss of paclitaxel. Kelsch et al.^12^ used a shake test to measure the adherence of the dry coating. The balloons were inflated and shaken in an empty vial. Additionally, the loss of paclitaxel was measured during passage through a blood-filled hemostatic valve and guiding catheter, and during one minute residence in stirred blood. The quantification of drug loss was performed with HPLC. Seidlitz et al.^13^ and Kempin et al.^14^ used a polymethacrylate model and gel cylinders to examine drug transfer. The DCB was pushed through the model and then inserted into the gel cylinder and expanded against the gel with 8 atm for one minute. For quantification, the residual substance fraction on the surface of the balloon, the substance fraction transferred to the gel, and the fluorescence intensity were measured against two standard calibration curves using a fluorescence reader and HPLC. Kaule et al.^15^ measured the drug-transfer to the vessel wall and residual drug concentration on the balloon surface in a vessel model with a silicone test tube on the distal end of the test path. After the DCB was pushed through the model to the silicone test tube, the balloon was inflated with 7 bar for 30 s. For quantification, HPLC was used. Brandt-Wunderlich et al.^16^ inserted a DCB into a porcine carotid artery in a vial with saline solution. The balloon was inflated and the pressure was held for 30 s. Afterwards, the balloon was deflated and removed from the artery and the vial. The quantification was performed with HPLC. All proposed methods treat the particle loss by handling during angioplasty as a black box. The concentration of the drug was measured before and after stress on the balloon surface with HPLC. The procedure of angiography can differ greatly and depends, for example, on the target region and the experience of the physician. Furthermore, only small groups of commercial DCBs were examined. Additionally, only a few publications compared different DCB types to each other. The present study allows a systematic evaluation of commonly used DCBs with regard to handling errors (e.g. careless handling when pulling off the protective cap or inserting the catheter into the sheath, frequent pulling back or forceful pushing, unfavorable path to the target region) or target regions that are difficult to reach, which may cause side effects like varying effective paclitaxel doses.

The study has some limitations. First, it is an in vitro test series without pulsating fluid system. The solubility of the particles can have an impact on the result although paclitaxel is hardly soluble in water^29^. In the inflation status, the vulnerability of drugs on the DCB that was examined by abrasion with a robotic arm can be significantly different from the transfer of drugs to the vessel wall. There are many other factors such as compliance of the DCBs (easy to contact the vessel wall), hydrophilic and lipophilic properties of excipients on each DCBs (easy to transfer to endotherial cell) and blood flow (solubility in blood). Therefore, higher vulnerability of drugs in inflation status estimated in this study is not equal to higher transfer of drugs to the vessel wall. However, the method is suitable to understand the influence of friction on the catheter sheath and vessel wall. Furthermore, the test series were carried out at room temperature. Further studies are required to find more evidence of connections between the clinical results and material properties of the DCBs.

In conclusion, the structures and compositions of the DCBs are different, resulting in different responses to frictional force. Particle loss by handling during angioplasty leads to different paclitaxel doses at the target regions for same DCB types. There are DCB types that are significantly more susceptible to drug loss. These properties can be the cause of side effects, which may explain variations in studies regarding the therapeutic outcome for the DCBs used.

## Materials and methods

### Experimental setup

**Figure 5:**
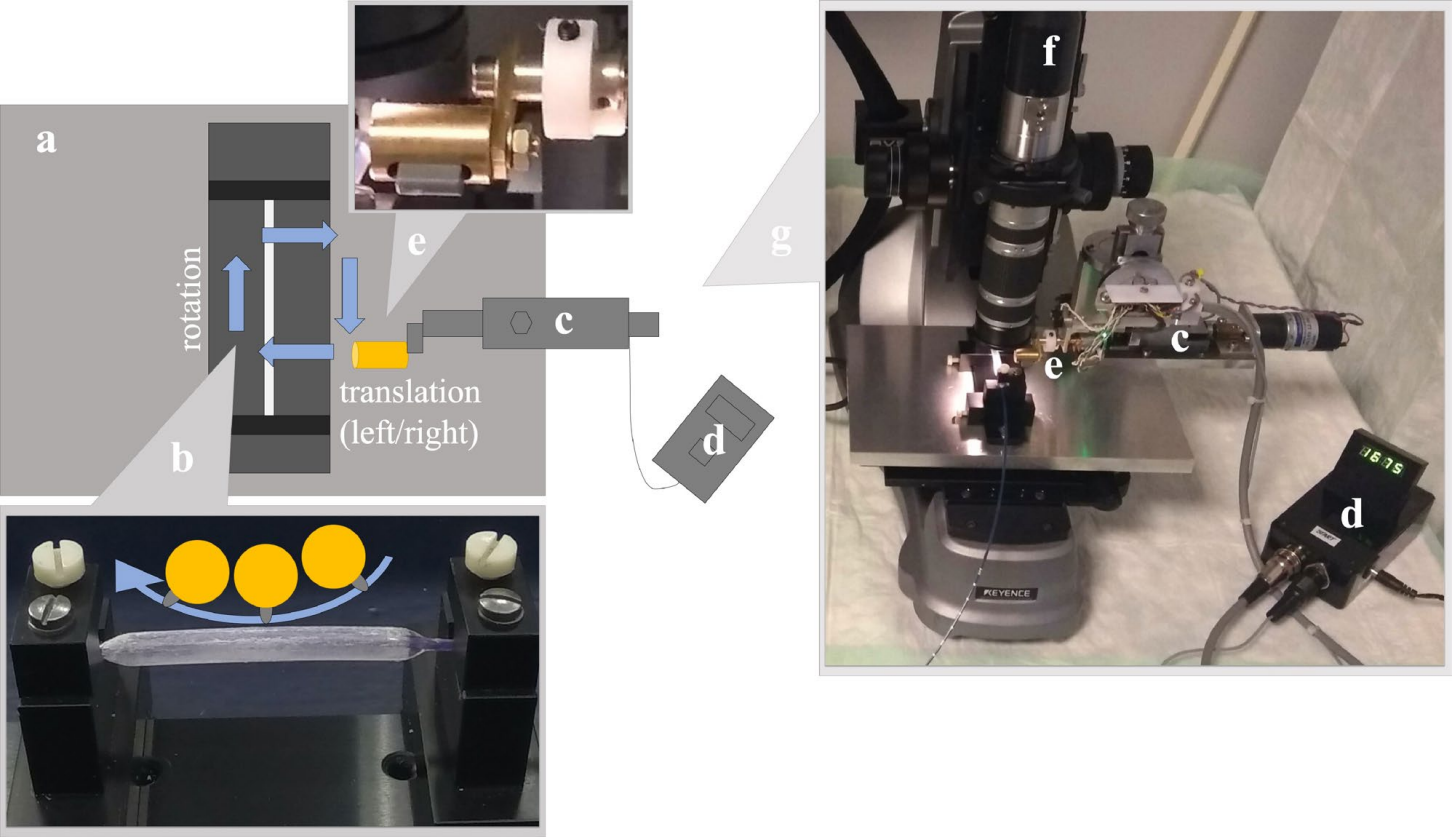
Experimental setup: (**a**) balloon holder (top view) with (**b**) illustration of the abrasion process, (**c**) robotic arm with (**d**) display of the load cell, (**e**) abrasion blade in close-up, (**f**) reflected light microscope and (**g**) photo of the experimental setup.

**Figure 6:**
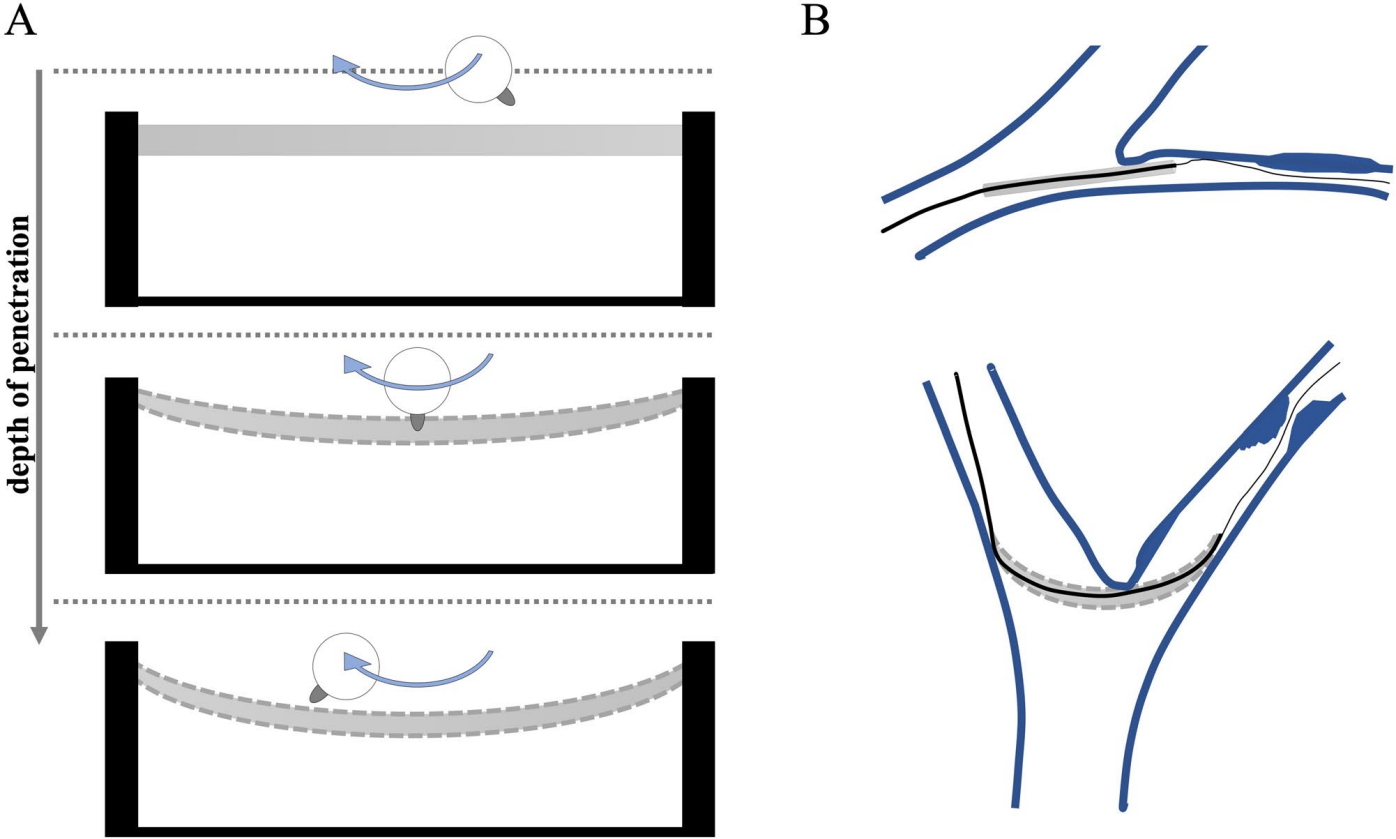
Schematic description of the procedure: (**A**) the depth of penetration and therefore also the contact force were gradually increased by reducing the height between balloon surface and abrasion blade by 0.5 mm after each cycle. (**B**) Illustration of a possible abrasion process in the vessel system. A catheter with an attached deflated balloon is advanced over a guide-wire along a sharp curve to the target area (narrowed or obstructed blood vessel) in the vascular system. The method should make the properties of DCB types comparable with one another under the same conditions. The realistic and tissue-equivalent implementation of an abrasion process in the vascular system is not the aim of this method.

The study included eight different commercially available DCB types for angioplasty with a length of 40 mm and a diameter of 5 mm (see Table 3). A reflected light microscope (VHX-500FD, KEYENCE Deutschland GmbH, Germany) with a polarizing filter (OP-35415, KEYENCE Deutschland GmbH, Germany) was provided with a robotic arm (see Fig. 5) intended to cause a reproducible and systematic stress on the balloon surface. The robot arm was made of brass with a motor mounting made of aluminum. A holding device made of polyvinyl chloride (PVC) made it possible to fix the balloon with nylon screws. Microcontrollers controlled a geared DC motor (V-TEC 6V, CN), which moved the robot arm in a circular motion over the balloon surface with a PVC abrasion blade (see Fig. 6A). This corresponds to a grinding movement of the DCB into the shaft and along a sharp curve in the vascular system (see Fig. 6B). A fine gear with a pitch of 0.1 mm allowed precise adjustment of the height between the balloon surface and the abrasion blade. A load cell measured the maximum contact force between the abrasion blade and the balloon surface.

### Measurements

The particle loss of a balloon (n = 3) including a guidewire was evaluated for deflated and inflated statuses (two series of measurements for a total of 24 DCBs). The balloons were fixed in the holding device with nylon screws. Some balloons had a coating in form of stripes, so the intact drug layer had to be oriented upwards in the direction of the abrasion device. In a series of measurements, the contact force was gradually increased by reducing the height between balloon surface and abrasion blade by 0.5 mm after each cycle. After each cycle, microscopic images were recorded at magnifications of 20, 30, 50, 100, 150 and 200×. A series of measurements was completed when the blade reached a depth of penetration of 4.50 mm (deflated) and 2.50 mm (inflated). For the inflated status, the inflation pressure specific for each DCB was used to reach the nominal diameter of 5 mm (compare with Table 3). The pressure was constant throughout the measurement series.

### Qualitative evaluation

The microscopic images were evaluated qualitatively with an assessment of particle loss in three categories: (0) no or hardly any abrasion of the drug/excipient in the deflated and inflated statuses, (1) no or hardly any abrasion of the drug/excipient in the deflated status and clear abrasion of the drug/excipient in the inflated status, and (2) clear abrasion of the drug/excipient in the deflated and inflated statuses.

### Quantitative evaluation

Furthermore, a quantitative measurement of drug loss was carried out via image processing, using the images at 50× magnification. To correct for shifts, all images of each series of DCB type and inflated/deflated status were aligned (registered) and cropped to the common overlapping area. Then, the images were converted to gray scale images by extracting the lightness channel after conversion from RGB to the Lab color space. Equalizing each gray scale image helped mitigating potential illumination differences. Residual reflections not already suppressed by the polarizing filter were filtered from the images by enforcing each pixel value in a series to be monotonously declining. Simple value thresholds removed remaining low-value reflections in the base material. The absolute loss numbers were determined by calculating pixel-wise differences, discretizing the difference values and counting the number of pixels of each discrete bin. Finally, the percentages of loss were calculated with respect to the number of non-zero pixels of the first image of each series.

The software SPSS Statistics version 26 (IBM, Armonk, USA) was used for statistical evaluation. The DCB types were compared using Independent-Samples Kruskal–Wallis Test with post hoc Dunn’s pairwise tests and Bonferroni corrections. Effect sizes (r)^30^ were calculated by dividing the standardized test statistics (z score) by the square root of the total observations, where r < 0.3, 0.3 ≤ r ≤ 0.5 and r > 0.5 denote small, medium, and large effect sizes, respectively. The level of significance was set to p < 0.05.

## Supplementary information

Supplementary Figures
