# Author Correction: Systematic evaluation of particle loss during handling in the percutaneous transluminal angioplasty for eight different drug-coated balloons

**DOI:** 10.1038/s41598-021-85117-5

**Published:** 2021-03-10

**Authors:** Andreas Heinrich, Martin S. Engler, Felix V. Güttler, Christian Matthäus, Jürgen Popp, Ulf K.-M. Teichgräber

**Affiliations:** 1 Department of Radiology, Jena University Hospital-Friedrich Schiller University, Am Klinikum 1, 07747 Jena, Germany; 2 Leibniz Institute of Photonic Technology, 07745 Jena, Germany; 3 Institute of Physical Chemistry & Abbe Center of Photonics, Friedrich Schiller University Jena, 07743 Jena, Germany

Correction to: *Scientific Reports* 10.1038/s41598-020-74227-1, published online 14 October 2020

This Article contains errors.

In Table 3, the Manufacturer for ‘Elutax 3’,

“Aachen Resonance GmbH”

should read:

“AR Baltic Medical UAB”

