# Supplementary material for: Systematic evaluation of particle loss during handling in the percutaneous transluminal angioplasty for eight different drug-coated balloons
Source: Sci Rep. 2020 Oct 14;10:17220. doi: 10.1038/s41598-020-74227-1 (PMC7560869; doi:10.1038/s41598-020-74227-1)
Supplement: Supplementary file 1 — Supplementary Figures [file 41598_2020_74227_MOESM1_ESM.pdf]

Systematic evaluation of particle loss during handling in the percutaneous transluminal angioplasty for eight different drug-coated balloons

Andreas Heinrich[1*], Martin S. Engler[1], Felix V. Güttler[1],
Christian Matthäus[2], Jürgen Popp[2], and Ulf K-M. Teichgräber[1]

[1]Department of Radiology, Jena University Hospital –
Friedrich Schiller University, 07747 Jena, Germany

[2]Leibniz Institute of Photonic Technology, 07745 Jena, Germany

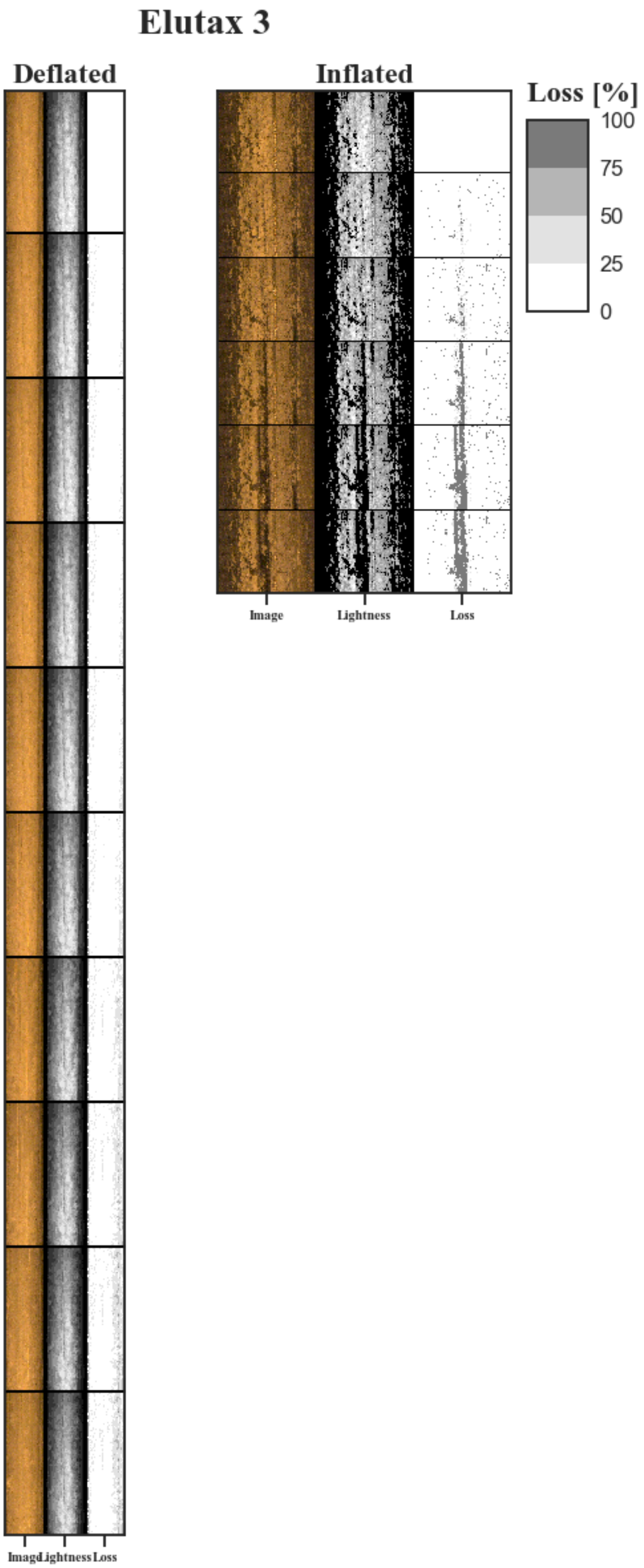

Fig. S1 Microscopic images recorded during the abrasion process for the deflated (left) and inflated (right) DCB (Eluatax 3). The images were converted to gray scale images by extracting the lightness channel. The absolute loss numbers were determined by calculating pixel-wise differences, discretizing the difference values and counting the number of pixels of each discrete bin. The percentages of loss were calculated with respect to the number of non-zero pixels of the first image of each series.

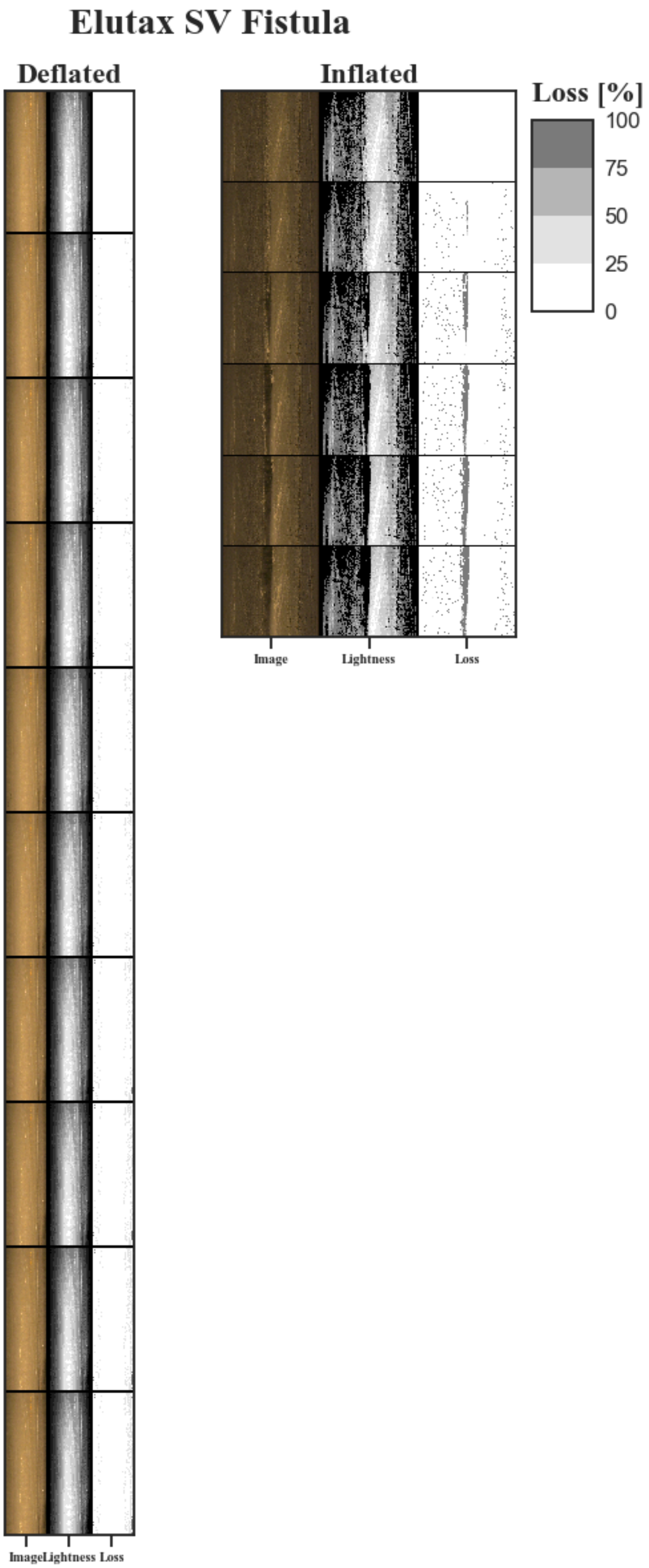

Fig. S2 Microscopic images recorded during the abrasion process for the deflated (left) and inflated (right) DCB (Eluatax SV Fistula). The images were converted to gray scale images by extracting the lightness channel. The absolute loss numbers were determined by calculating pixel-wise differences, discretizing the difference values and counting the number of pixels of each discrete bin. The percentages of loss were calculated with respect to the number of non-zero pixels of the first image of each series.

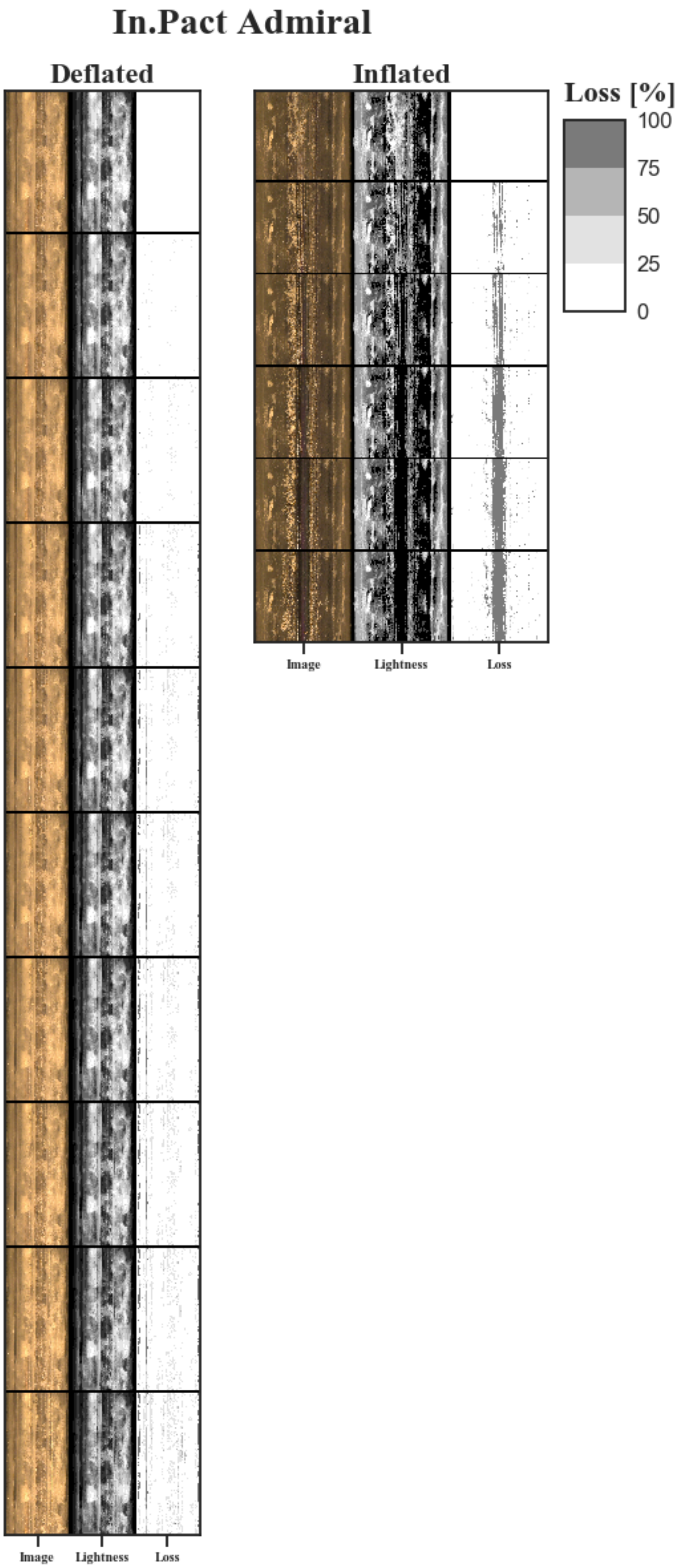

Fig. S3 Microscopic images recorded during the abrasion process for the deflated (left) and inflated (right) DCB (In.Pact Admiral). The images were converted to gray scale images by extracting the lightness channel. The absolute loss numbers were determined by calculating pixel-wise differences, discretizing the difference values and counting the number of pixels of each discrete bin. The percentages of loss were calculated with respect to the number of non-zero pixels of the first image of each series.

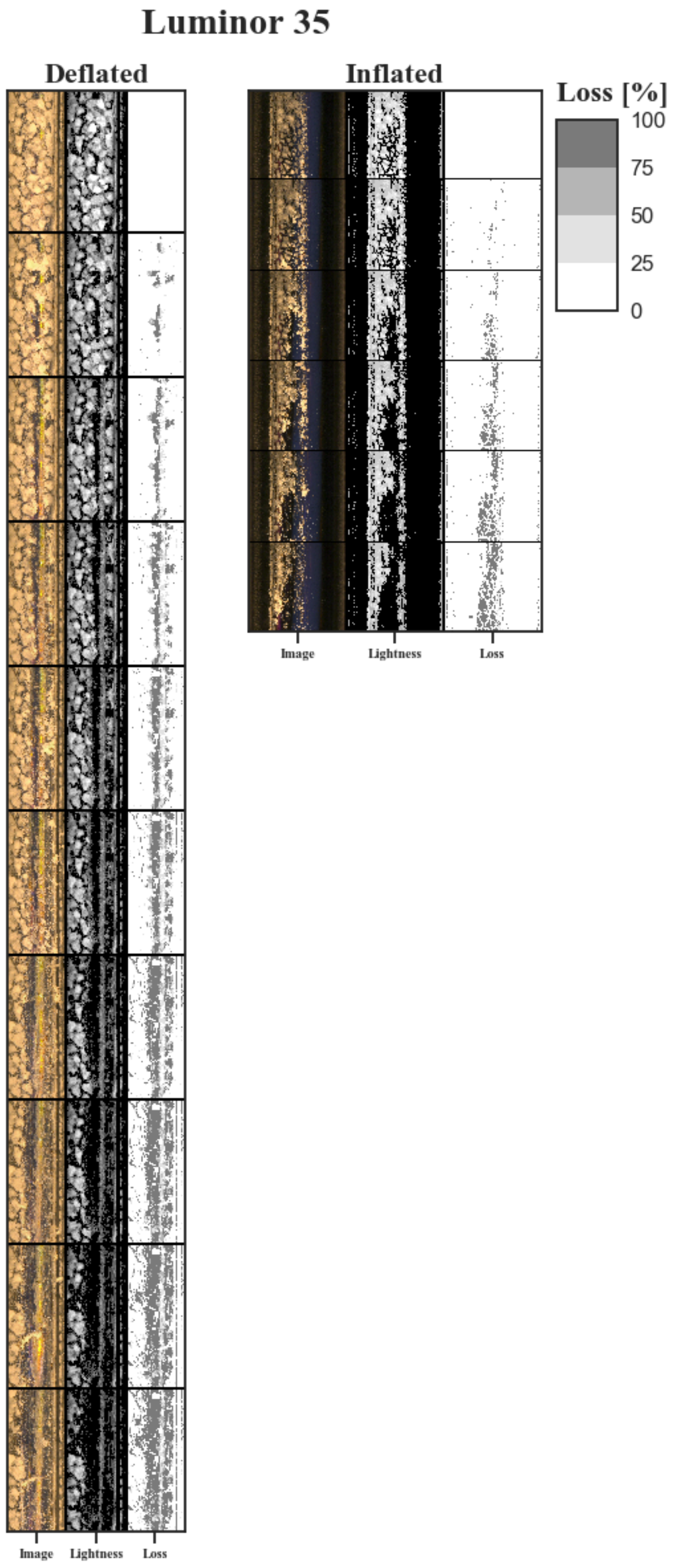

Fig. S4 Microscopic images recorded during the abrasion process for the deflated (left) and inflated (right) DCB (Luminor 35). The images were converted to gray scale images by extracting the lightness channel. The absolute loss numbers were determined by calculating pixel-wise differences, discretizing the difference values and counting the number of pixels of each discrete bin. The percentages of loss were calculated with respect to the number of non-zero pixels of the first image of each series.

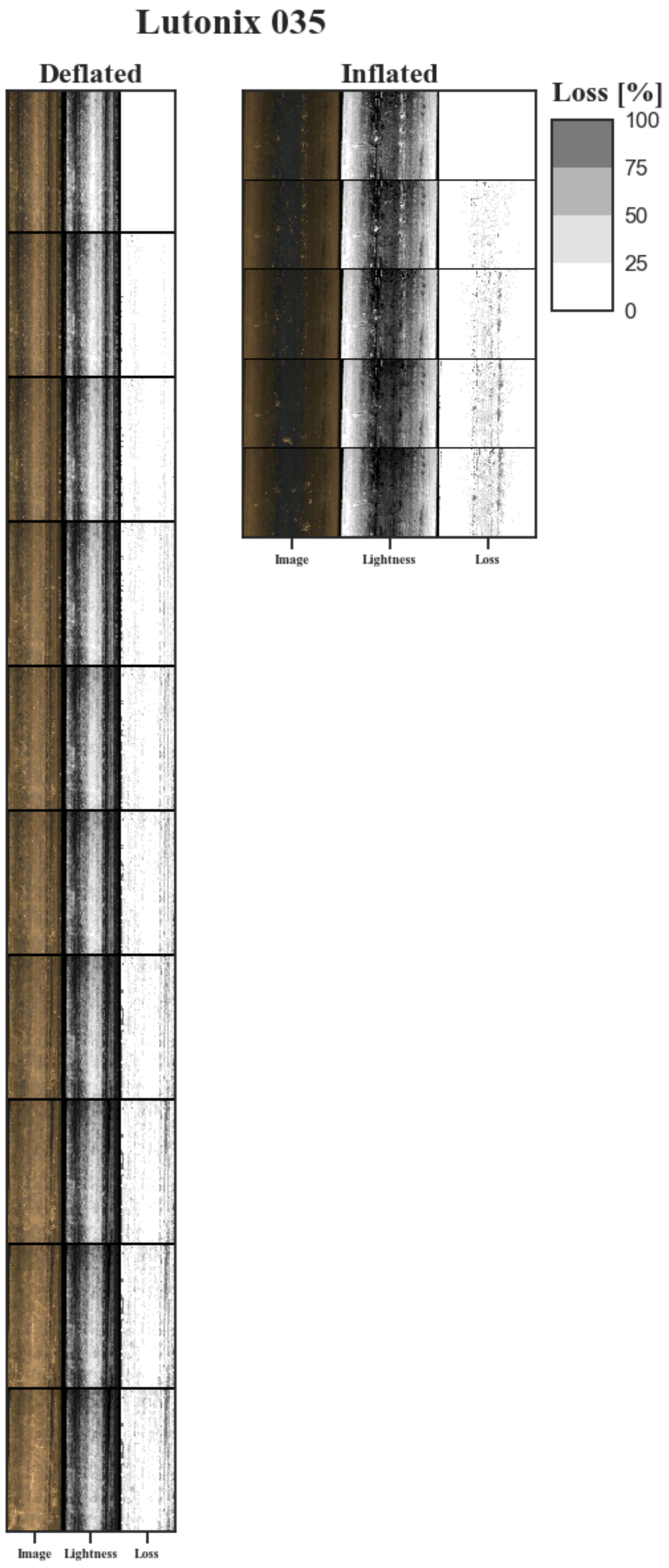

Fig. S5 Microscopic images recorded during the abrasion process for the deflated (left) and inflated (right) DCB (Lutonix 035). The images were converted to gray scale images by extracting the lightness channel. The absolute loss numbers were determined by calculating pixel-wise differences, discretizing the difference values and counting the number of pixels of each discrete bin. The percentages of loss were calculated with respect to the number of non-zero pixels of the first image of each series.

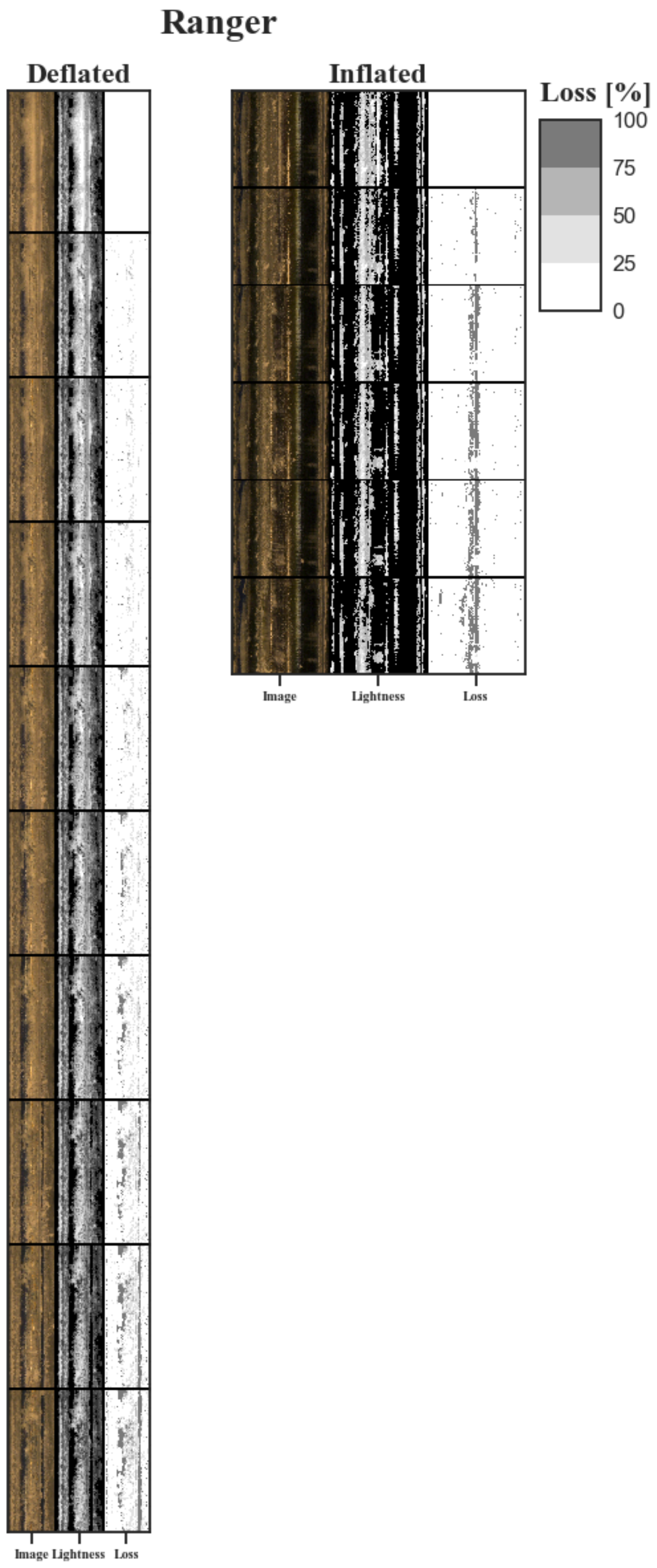

Fig. S6 Microscopic images recorded during the abrasion process for the deflated (left) and inflated (right) DCB (Ranger). The images were converted to gray scale images by extracting the lightness channel. The absolute loss numbers were determined by calculating pixel-wise differences, discretizing the difference values and counting the number of pixels of each discrete bin. The percentages of loss were calculated with respect to the number of non-zero pixels of the first image of each series.

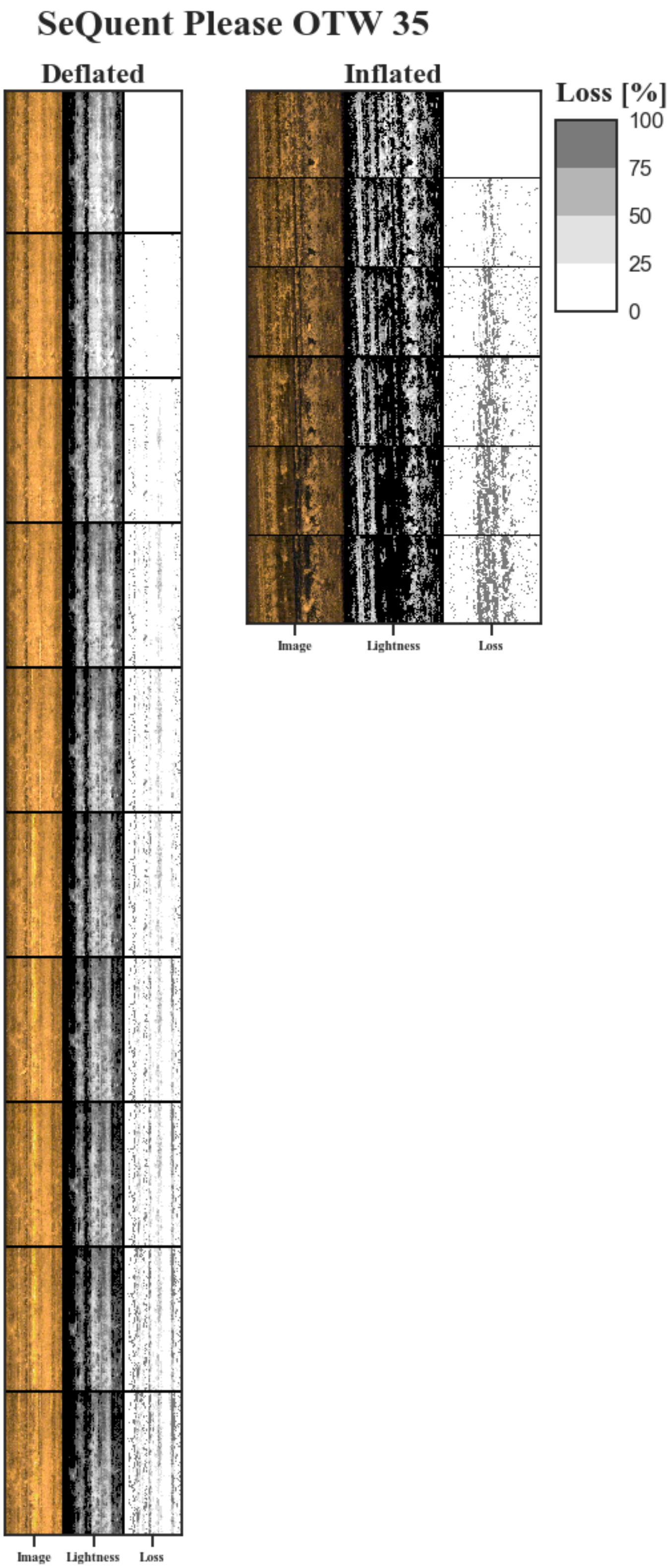

Fig. S7 Microscopic images recorded during the abrasion process for the deflated (left) and inflated (right) DCB (SeQuent Please OTW 35). The images were converted to gray scale images by extracting the lightness channel. The absolute loss numbers were determined by calculating pixel-wise differences, discretizing the difference values and counting the number of pixels of each discrete bin. The percentages of loss were calculated with respect to the number of non-zero pixels of the first image of each series.

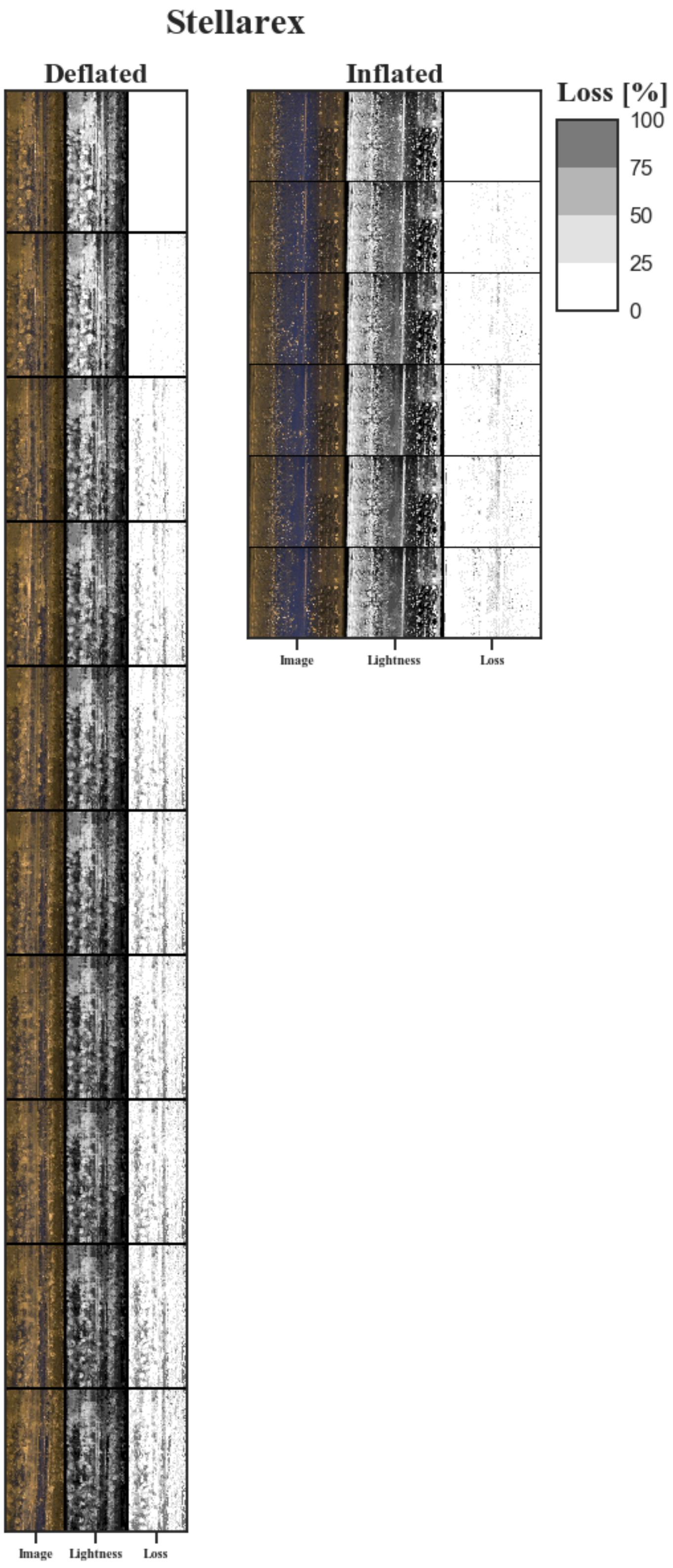

Fig. S8 Microscopic images recorded during the abrasion process for the deflated (left) and inflated (right) DCB (Stellarex). The images were converted to gray scale images by extracting the lightness channel. The absolute loss numbers were determined by calculating pixel-wise differences, discretizing the difference values and counting the number of pixels of each discrete bin. The percentages of loss were calculated with respect to the number of non-zero pixels of the first image of each series.
